# Boosting Flame Retardancy of Polypropylene/Calcium Carbonate Composites with Inorganic Flame Retardants

**DOI:** 10.3390/ma17184553

**Published:** 2024-09-16

**Authors:** Antonio Benjamim Mapossa, Erick Gabriel Ribeiro dos Anjos, Uttandaraman Sundararaj

**Affiliations:** Department of Chemical and Petroleum Engineering, University of Calgary, 2500 University Drive NW, Calgary, AB T2N 1N4, Canada; mapossabenjox@gmail.com (A.B.M.); erick.anjos@unifesp.br (E.G.R.d.A.)

**Keywords:** polypropylene, calcium carbonate, flame retardant, zinc borate, magnesium hydroxide

## Abstract

This study investigates the effects of inorganic flame retardants, zinc borate, and magnesium hydroxide, on the thermal, morphological, flame retardancy, and mechanical properties of polypropylene (PP)/calcium carbonate composites for potential construction industry applications. Polypropylene/calcium carbonate (50 wt.%) composites containing 5 and 10 wt.% flame retardants were prepared using a batch mixer, followed by compression moulding. The results demonstrated enhanced thermal stability, with the highest char residue reaching 47.2% for polypropylene/calcium carbonate/zinc borate (10 wt.%)/magnesium hydroxide (10 wt.%) composite, a notably strong outcome. Additionally, the composite exhibited an elevated limited oxygen index (LOI) of 29.4%, indicating a synergistic effect between zinc borate and magnesium hydroxide. The proposed flame retardancy mechanism suggests that the flammability performance is driven by the interaction between the flame retardants within the polypropylene/calcium carbonate matrix. Magnesium hydroxide contributes to smoke suppression by releasing water, while zinc borate forms a protective glassy foam that covers the burning surface, promoting char formation and acting as a physical barrier to heat transmission and fire spread. Scanning electron microscopy confirmed good dispersion of the additives alongside calcium carbonate within the polymer matrix. Despite the addition of up to 10 wt.% flame retardants, the composites maintained high-notched impact strength.

## 1. Introduction

Polypropylene (PP) is one of the most important thermoplastic polymers in use in various applications, including in the construction industry. It is easy to process, and it has relatively high mechanical properties, great recyclability, and low cost, which make it an excellent material for everyday use [1]. However, the principal concern is related to its flammability which very often limits its wider application fields including the construction industry [2]. Therefore, to reduce the flammability of polypropylene, several fillers and additives known as flame retardants can be added to the PP and these can act in various modes [3,4,5,6].

Nowadays, several intumescent flame retardants, including halogenated and phosphorus nitrogen materials, are still being incorporated into the polymer matrix to enhance its flame retardancy. However, there are substantial limitations concerning halogenated flame retardants because, during thermal decomposition, they release toxic gases and smoke that are hazardous to people and the environment [2]. Inorganic flame retardants, such as magnesium hydroxide, aluminum hydroxide, and zinc borate, are the most common additives used to improve the flame retardancy of polypropylene composites-based calcium carbonate, whilst preserving their mechanical properties. Recently, a study demonstrated that to reach a high performance of flame retardancy of polymer composites, a combination of flame retardants (i.e., inorganic flame retardants like magnesium hydroxide (MH) and zinc borate (ZB)) need to be added to polypropylene to obtain a synergistic effect, which is superior to the effect of individual flame retardants on flammability properties [7].

Zinc borate, containing different contents of zinc and boron oxides, is a multifunctional flame retardant widely employed in various polymer applications to enhance the flame retardancy of several polymers, including polypropylene [8,9,10,11,12,13,14]. This inorganic flame retardant is a non-toxic flame retardant and exhibits high thermal stability [12,13]. Furthermore, studies have demonstrated that zinc borate during thermal decomposition produces an improved glassy foam on the surface of the polymer matrix capable of insulating the polymer from the burning zone [6,12]. It also acts as an intumescent flame-retardant compound by forming carbonaceous char [15].

Magnesium hydroxide (MH) is one of the most common flame retardants for polyolefins like polypropylene, with low toxicity used to improve flame retardancy and suppress smoke [16,17]. Therefore, MH is widely used in industrial applications due to its low cost, high thermal stability, and thermal isolation effect [18,19,20]. Zaghloul et al. [18] (2017) evaluated the influence of magnesium hydroxide on the mechanical properties of polyolefins (HDPE). The results demonstrated that the reinforced materials presented substantial influence on the mechanical properties of the HDPE composites containing MH with concentrations from 5 to 50 wt.%. Furthermore, a study conducted by Jiao et al. [17] showed that magnesium hydroxide remarkably improved the flame retardancy of EVA copolymer, demonstrated by an enhancement of LOI values from 20% for neat EVA to nearly 38% for EVA/MH composites.

In the construction industry, besides the enhanced thermal stability and flame retardancy, the enhanced mechanical properties of polymer materials are also required. These properties can be achieved by adding fillers, such as calcium carbonate (CaCO_3_), to the PP matrix. In this context, CaCO_3_ is the most used filler in polymer applications because it is inexpensive and widely available. Consequently, CaCO_3_ is largely used to reduce the final cost of the polymer products [21], as well as enhance the polymer processability [22]. This ceramic material also changes the physical properties of the polymer [23,24]. Furthermore, the addition of CaCO_3_ to the polypropylene enhances some specific characteristics, including heat deflection temperature stiffness and dimensional stability of the polymer.

Some studies done by [22,25] have reported that calcium carbonate increases the mechanical properties, particularly, the impact strength of polymer. The filler helps to improve conversion efficiency as it suppresses melt flow instabilities that lead to melt fracture during extrusion, such as when converting polyolefin to sheet [22]. Therefore, PP/CaCO_3_ masterbatches are extensively utilized in processes, such as the extrusion of polypropylene film and sheet materials [22], to achieve a reduction in product cost.

The literature reports the preparation of polypropylene-based flame retardants such as ammonium polyphosphate, melamine polyphosphate, and inorganic flame retardants [15,26,27,28]. One study conducted by Li et al. [29] reports the synergistic effect of intumescent flame retardant and nano-CaCO_3_ on the flammability properties of polypropylene. Unlike the current work, Li et al. [29] used a low amount of calcium carbonate (1 to 5 wt.%) and intumescent flame retardants ammonium polyphosphate (APP), melamine (MEL), and pentaerythritol (PER) in amounts varying from 19 to 40 wt.%.

However, to the best of our knowledge, the fabrication of polypropylene based on high loading of CaCO_3_ (i.e., 50 wt.%) reinforced with magnesium hydroxide and zinc borate to create a synergistic effect, has not yet been investigated and is therefore undertaken here. Furthermore, we study the effect of inorganic flame retardants (zinc borate and magnesium hydroxide) on the thermal, morphological, flame retardancy, and mechanical properties of polypropylene (PP)/calcium carbonate composites to be applied in the construction industry. A batch mixer is used to prepare PP/CaCO_3_ (50 wt.%) composites containing 5 and 10 wt.% of flame retardants, followed by sample preparation using compression moulding. A flame retardancy mechanism of zinc borate combined with magnesium hydroxide in the PP/CaCO_3_ composites is proposed. The results generated from this work allow for the development of cost-effective, processable, flame retardancy materials based on polypropylene/calcium carbonate composites, reinforced with inorganic flame retardants with suitable thermal, flammability, and mechanical properties to be applied in the construction industry.

## 2. Materials and Methods

Polypropylene resin (grade Homopolymer H5104) with a density of 0.930 g/cm^3^ and melt flow index (MFI) of 4.0 g/10 min (2.16 kg at 230 °C) was provided by Heartland Polymers (Calgary, AB, Canada). Inorganic flame retardants, zinc borate (2ZnO·3B_2_O_3_·3.5H_2_O; Firebrake^®^ 500) was provided by U.S. Borax Firebrake^®^ and magnesium hydroxide was obtained from Sigma-Aldrich. Calcium carbonate (particle size of 44 µm) was provided by Graymont, (Canmore, AB, Canada).

Before the fabrication of PP composites, calcium carbonate was dried in an oven at 60 °C for 72 h. The polypropylene containing a fixed quantity of calcium carbonate (50 wt.%) and 5 wt.% and 10 wt.% of inorganic flame retardants (ZB and MH) were fabricated using a Haake Rheomix batch mixer (Thermo Fischer Scientific, Waltham, MA, USA), working with speed of rotors of 100 rpm at 220 °C for a total mixing time of 12 min. Parameters such as torque and melt temperature were constantly monitored throughout the polymer composite compounding process. Table 1 lists the neat PP and different polypropylene/calcium carbonate composites with magnesium hydroxide and zinc borate at different concentrations.

The viscosity of the composites and neat PP was evaluated in the steady-state regime using a Dynisco Rheometer (model Galaxy V) capillary rheometer, following ASTM D3835-16. A silicon carbide die with a diameter of 1.25 mm (L/D ratio of 20) was employed. The analyzed shear rates ranged from 10 to 1000 s^−1^ at a temperature of 220 °C to match the processing temperature. Data corrections were applied using the Weissenberg-Rabinovitch method. No rheological phenomena, such as melt fracture or shark skin, were observed during the analysis.

Morphological evaluation was conducted using scanning electron microscopy (SEM) on cryofractured surfaces of the composites. The analyses were performed with an FEI Quanta 250 FESEM microscope. All surfaces were platinum-coated using a sputter-coater. Additionally, energy-dispersive X-ray spectroscopy (EDS) was carried out on the SEM for high-magnification images, targeting characteristic elements of each filler/additive (Ca, Mg, and Zn) with an accumulation time of 5 min.

Dynamic scanning calorimetry (DSC) analyses of the composites were conducted using a TA model Q2000 DSC instrument under a nitrogen gas atmosphere. The temperature range evaluated was from room temperature 25 °C to 250 °C, with a heating rate of 5 °C min^−1^. The procedure included heating, cooling, and a second heating cycle. The crystallinity degree (X_c_ (%)) of the PP matrix was calculated from the melting enthalpy (ΔH_m_) of both heating data using Equation (1):

(1) $$X_{c}\left(\%\right)=\frac{{∆H}_{m}}{{∆H}_{PP}^{0}\left(\varphi \right)}\times 100$$

where φ represents the weight fraction of PP in the composition, and ΔH°_PP_ is the theoretical melting enthalpy of a 100% crystalline PP (~207 J/g) [30,31,32,33,34,35,36]. 

The thermogravimetric analysis model (TGA Q500 V20.10 Build 36) was used to evaluate the thermal stability of polypropylene-based calcium carbonate composites reinforced with flame retardants and their char residues. Therefore, the TGA conditions used to run the samples included a range of temperatures, from ambient temperature to 900 °C, with a nitrogen flow rate of 50 mL min^−1^ using a heating rate of 10 °C min^−1^.

The flammability properties of neat PP and samples based on PP/CaCO_3_ composites reinforced with zinc borate and magnesium hydroxide were investigated using the limiting oxygen index (LOI) equipment Model AT-P6012A (Amade Technology Co., Limited, Hong Kong, China). The limiting oxygen index represents the minimum oxygen concentration in oxygen and nitrogen mixtures needed to sustain the flame of the PP composite materials, at ambient temperature at 3 min of the burning time of the material. The LOI measurement was conducted based on the standard ASTM D2863 protocol.

The impact strengths of neat polypropylene and polypropylene composites were obtained using the Notched Izod impact equipment model Tinius Olsen Mode Impact 104 tester (Tinius Olsen, Horsham, PA, USA). The test was performed based on the recommended standard ISO 180. For the impact test, the notched moulded samples were prepared using a Qualitest QC-640A impact specimen angle cutting device (Qualitest, Lauderdale, FL, USA) to achieve a *v-notch* for the Izod impact test. The Izod Impact strengths (kJ/m^2^) correspond to an average of the five samples for each composition.

The tensile measurements of neat PP and PP-based calcium carbonate with flame retardants were conducted using the equipment model, INSTRON 5965, Norwood, MA, USA, with a maximum capacity of force of 5 KN. The test was performed according to standard ASTM D638. The values correspond to an average of the four samples for each composition.

The fire test was conducted to assess the char structure and its expansion of PP/CaCO_3_ composites reinforced with inorganic flame retardants (ZB and MH) when these samples were burned via pyrolysis in air at 900 °C for 20 min to guarantee the entire sample burned [31,32]. The samples were cooled in the pyrolysis oven for 120 min to prevent thermal shocks to the char. Finally, the measurement of the char expansion degree was done at 10 points using a digital Vernier calliper, and the average char expansion degree was registered. The char expansion degree (C (%)) was calculated using Equation (2).

(2) $$C\left(\%\right)=\frac{{d_{2}-d}_{1}}{d_{1}}\times 100$$

Here, *d*_1_ is the initial thickness of the samples before pyrolysis test and *d*_2_ is the final thickness of the expanded char after pyrolysis test.

## 3. Results and Discussion

### 3.1. Processability and Rheological Properties

**Figure 1:**
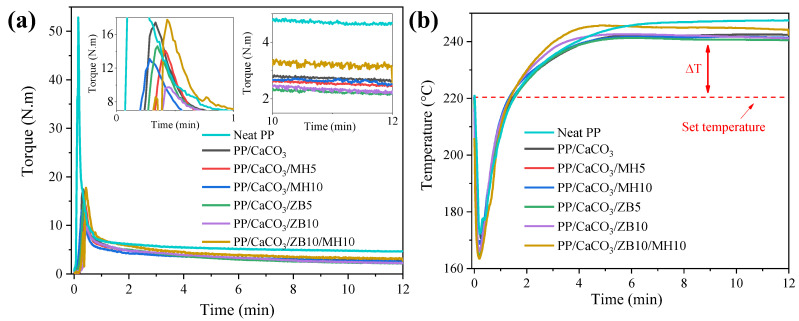
(**a**) Torque of neat PP and PP/CaCO_3_ composites with flame retardants in the function of time and (**b**) Temperature profile of processing of neat PP and PP/CaCO_3_ composites with flame retardants in the function of time.

The torque and temperature of the mixing process were controlled and measured during the processing of the composites (Figure 1). These parameters provide valuable insights into the behaviour of different compositions during processing. Initially, a peak in torque is observed within the first minute, corresponding to the mixing, polymer melting, and homogenization of the composite materials. This is followed by a plateau or steady-state torque value, achieved after two minutes, and mixing further ensures better dispersion and distribution of the fillers and is maintained until the end of the mixing process (12 min total).

The torque peak and equilibrium torque for neat PP were significantly higher than those observed for the composites. This difference can be attributed to two main factors. First, the lower density of PP results in a greater material volume for the same weight fed into the batch mixer. Additionally, the specific grade of PP used is designed for extrusion applications, and thus, has a low melt flow ratio of 4.0 g/10 min (2.16 kg at 230 °C). This indicates a high molecular weight and/or more and longer branches giving an increased number of polymer chain entanglements, which in turn leads to a higher viscosity. In contrast, the composites, which have a high content of fillers (e.g., 50 wt.% of CaCO_3_), experience a reduction in the volume of material within the chamber. This filler content not only decreases the number of total polymer chain entanglements per unit volume but also reduces the material’s volume in the mixer, both of which contribute to the lower torque observed for the composites.

Among the composites, it is noted that the composite with the highest filler content (70 wt.%-PP/CaCO_3_/ZB10/MH10) exhibits a slightly delayed torque peak and a marginally higher equilibrium torque. This delay behaviour can be attributed to the significantly lower polymer content in this composition, which takes longer to melt and incorporate the ceramic particles, forming a continuous matrix. Subsequently, more torque is required for mixing due to the elevated filler content. However, it is important to note that these variations are not significant from an application perspective, since the neat polymer requires a much higher torque to process alone.

During the first minute, the addition of solid materials at room temperature causes an instantaneous drop in the chamber temperature to around 165 °C. As the polymer melts and the materials are distributed and dispersed, the temperature stabilizes, reaching a plateau approximately 20 °C higher than the set temperature. This increase is due to viscous heating from the friction between polymer chains and between polymer and filler particles [33]. Most compositions exhibit similar behaviour, and the addition of inorganic flame retardants had a minimal effect on torque change compared to the base composition of PP and 50 wt.% of CaCO_3_.

**Figure 2:**
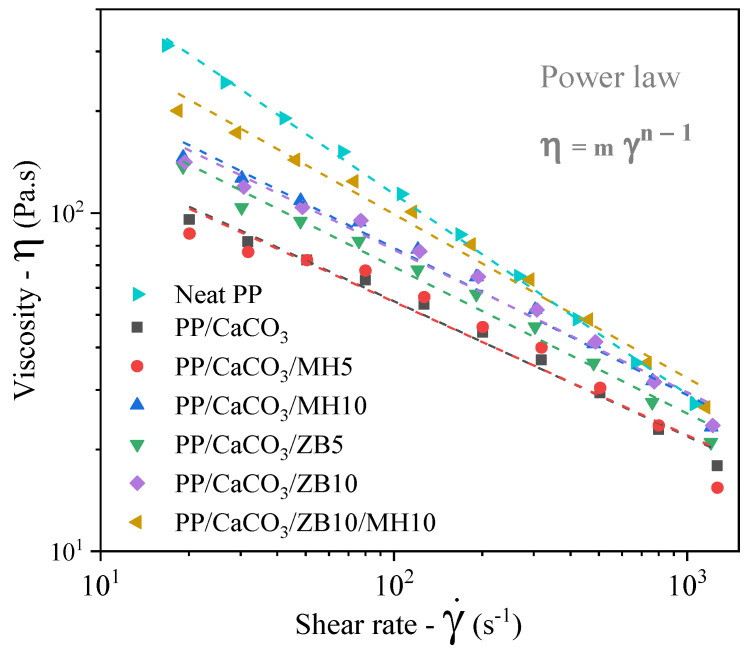
Viscosity in the function of shear rate of neat PP and PP/CaCO_3_ composites with flame retardants obtained by capillary rheometry.

The extrusion processing behaviour of the composites was evaluated from a viscosity perspective, characterized by a capillary rheometer within a shear rate range covering common extrusion process shear rates (10^2^ to 10^3^ s^−1^) (Figure 2) [34]. It is well-known that, aside from the high CaCO_3_ contents commonly used for automotive and housing polymer composites applications [34], improving the flammability properties of composites using traditional inorganic fillers often requires high flame-retardant content [2]. These elevated filler and additive contents may negatively impact some properties of polymer composites, such as mechanical properties and processability [2].

The viscosity results indicate that the addition of 50 wt.% CaCO_3_ exhibited a lubricant effect, reducing viscosity compared to neat PP. This effect can be understood from a microstructural perspective as a general reduction in the polymer chain entanglements per volume of material, caused by substituting part of the matrix volume with large CaCO_3_ particles that have low surface interaction with the PP matrix.

It is worth noting that while these observations align with the torque behaviour observed during the processing of these materials, they contrast with much of the existing literature on the rheological behaviour of PP/CaCO_3_ composites. Typically, studies report an increase in PP viscosity with the addition of CaCO_3_ due to the restriction of polymer chain mobility [34,35]. However, these behaviours are strongly correlated with the specific characteristics of the polymer and the shape of the filler particles, which differ from those in our study [35,36]. Complementary to those observations, Supaphol and Harnsiri [36] have reported no significant difference in steady-state viscosity at high shear rates of PP filled with different contents of coated CaCO_3_ particles measured by capillary rheometry. This may explain the low increase in the viscosity observed even for the maximum amount of filler.

When adding MH and ZB inorganic flame-retardant additives, a low but proportional increase in viscosity values was observed, with a more significant increase for ZB. For instance, PP/CaCO_3_/MH5 behaved similarly to PP/CaCO_3_, while PP/CaCO_3_/ZB5 exhibited significantly higher viscosity than PP/CaCO_3_. For most other compositions, the behaviour was similar; however, for the PP/CaCO_3_/ZB10/MH10 composite, the viscosity values approached those of the neat PP. These increments in viscosity for compositions with higher flame retardant contents could be related to the shape of the additive particles, their interaction with the polymer chains, and the composite morphology. Smaller additive particles than CaCO_3_ have more surface interaction with polymer chains, restricting chain mobility and increasing flow resistance. Additionally, their smaller size allows them to distribute in the matrix regions between large CaCO_3_ particles.

These parameters can be numerically observed from the fit data of the viscosity curves and modelled according to the power law coefficients (Table 2). Firstly, most linear fit parameters presented high accuracy (indicated by R^2^ values close to 0.98 for most compositions), with the highest value found for neat PP, as expected, due to its homogeneous morphology compared to composites. Secondly, the power law coefficients align with the observations, showing a higher consistency index for PP than for the composites, which increases with additive content for most composites. Concurrently, the pseudoplasticity index is lower for neat PP, indicating a higher shear-thinning effect, and is higher for composites, which have a higher volume fraction of solid particles. However, the addition of inorganic flame additives decreases the *n* value compared to the PP/CaCO_3_ composition in most cases, which could again be related to these additives’ interactions with the polymer matrix and composite morphology, as discussed in the following section.

### 3.2. Morphological Evaluation (SEM with EDS Analysis)

**Figure 3:**
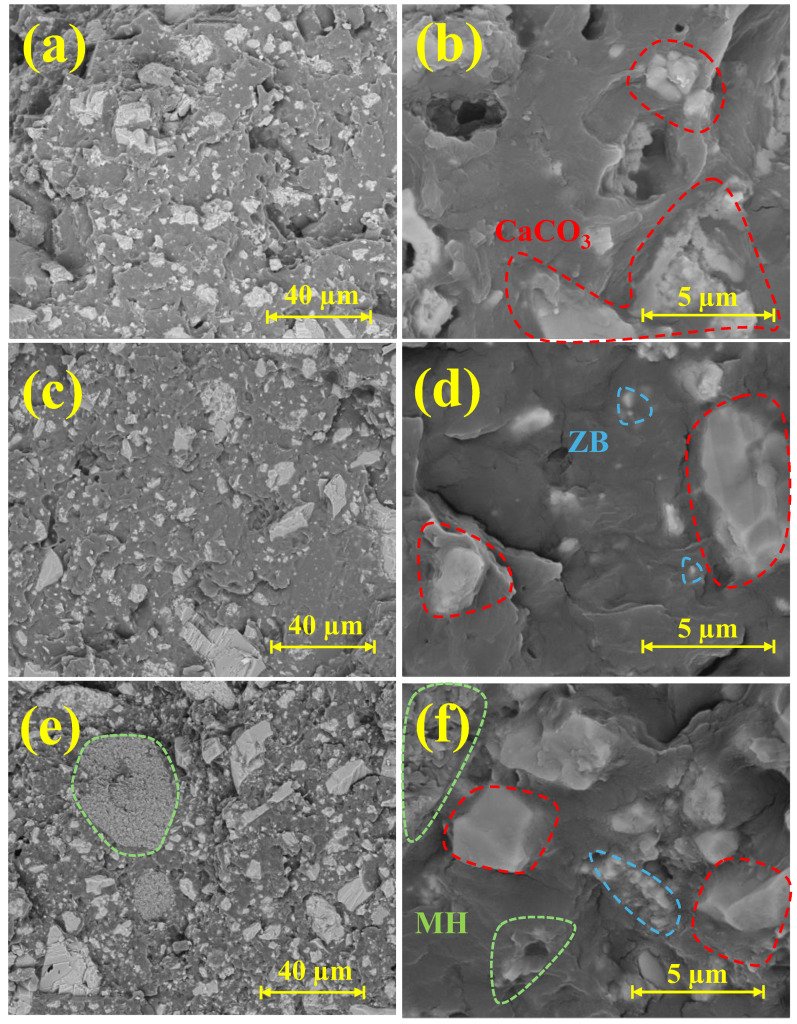
SEM micrographs of (**a**,**b**) PP/CaCO_3_; (**c**,**d**) PP/CaCO_3_/ZB10; and (**e**,**f**) PP/CaCO_3_/ZB10/MH10 composites.

The morphological evaluation was performed on the cryofracture surfaces of composite samples and was coupled with EDS analyses to identify each ceramic particulate material within the polymer matrix (Figure 3). The morphology of the PP/CaCO_3_ composites was homogeneously distributed throughout the PP matrix (Figure 3a,b). Subsequently, the PP/CaCO_3_/ZB10 morphology resembled that of PP/CaCO_3_, as expected, due to its composition. The major differences were observed through EDS analysis, which indicated the presence of Zn in smaller, more rounded particles attributed to ZB (Figure 3c,d). Finally, the PP/CaCO_3_/ZB10/MH10 composites displayed all three ceramic particles, with clearer distinctions between CaCO_3_ and ZB surfaces, and the morphology of MH. The MH particles were present among other particles in the matrix and formed large agglomerates in the PP composites (Figure 3c,d). This means that the addition of MH particles into the PP matrix results in the formation of some voids between the particles and the matrix and the formation of agglomerates in the PP composite, which is attributed to the low compatibility between them and the increased contact between particles.

### 3.3. Differential Scanning Calorimetry (DSC)

**Figure 4:**
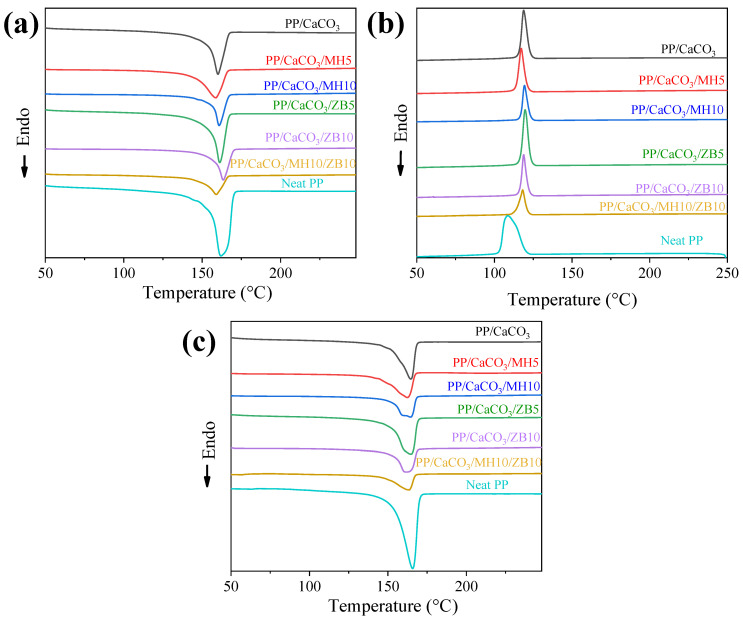
DSC curves of neat PP and PP/CaCO_3_ composites with zinc borate and magnesium hydroxide: (**a**) First heating curves, (**b**) second heating curves, and (**c**) cooling curves.

The DSC analysis results are summarized in Table 3 and Figure 4. Notably, for all composites, the peaks for melting and crystallization are smaller than those of neat PP. This is directly related to the lower volume fraction of PP in these respective samples.

According to the results for neat PP and PP/CaCO_3_ samples, the addition of 50 wt.% of CaCO_3_ did not significantly influence the polymer melting temperature (Tm). In addition, the crystallinity degree of the PP fraction in the PP/CaCO_3_ sample closely matched that of neat PP. This indicates that the melting behaviour is not significantly altered, and thus the service temperature of PP remains unchanged. However, during cooling, a different behaviour was observed with an increase of approximately 10 °C in the crystallization temperature. This increase is beneficial as it implies a reduction in processing time, with the composites solidifying/stiffening at a higher temperature, thereby reducing cooling time. This 10 °C shift is clearly observed in the cooling curves (Figure 4b). These results corroborate with previous literature studies [30,37].

The changes in behaviour observed for PP/CaCO_3_ compared to neat PP were consistently reproduced in the other composites. This similarity between different composites can be attributed to the higher content of CaCO_3_ compared to the additives, making it the dominant factor for this thermal behaviour. The only exception was the PP/CaCO_3_/ZB10/MH10 composite, which showed a significant reduction in crystallinity degree for both heating cycles. Considering that this composition has the maximum weight percentage of ceramic particles, this reduction can be explained by the blocking of the PP lamellae crystalline phase during crystal growth, consequently resulting in a lower crystallinity degree.

### 3.4. Thermogravimetric Analysis (TGA)

**Figure 5:**
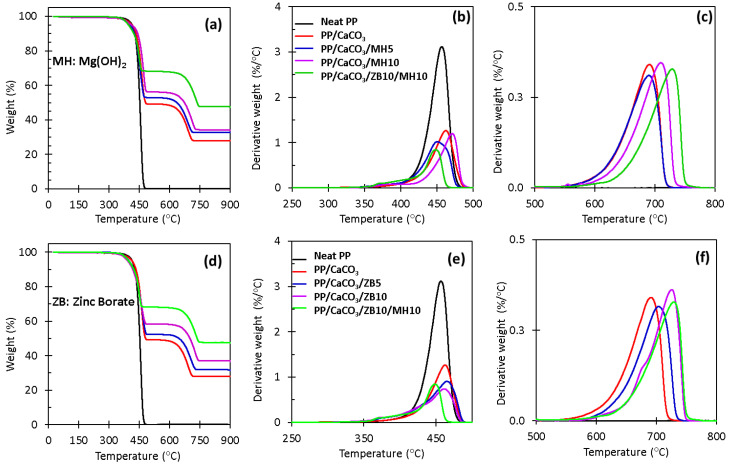
TGA curves of (**a**) neat PP and PP/CaCO_3_/MH composites (5 and 10 wt.% of MH); (**b**) DTG of neat PP and PP/CaCO_3_/MH composites; (**c**) DTG of PP/CaCO_3_ and MH showing the stability of calcium carbonate; (**d**) neat PP and PP/CaCO_3_/ZB (5 and 10 wt.% of ZB); (**e**) DTG of neat PP and PP/CaCO_3_/ZB composites; and (**f**) DTG of PP/CaCO_3_ and ZB showing the stability of calcium carbonate.

The effect of calcium carbonate and inorganic flame retardants on the thermal stability of PP was investigated using thermogravimetric analysis. This technique provides excellent information related to the flame retardancy properties (i.e., char residue) of the materials. TGA and DTG curves of neat PP and its composites reinforced with flame retardants are shown in Figure 5, and the corresponding TGA data are summarized in Table 4. The TGA results demonstrated that the trend of increasing decomposition temperature for polypropylene was observed as more calcium carbonate and flame retardants (FR) were added to the PP matrix. This indicates that the polypropylene chains had reduced flexibility in the presence of calcium carbonate and FR, and a strong polymer network enhanced the thermal stability of PP [38]. Based on DTG profiles for all samples, the decomposition temperature ranges are clearly visible. For neat PP, a simple one-step decomposition profile was observed from 340 to 480 °C, corresponding to the degradation of PP without any char residue formed.

Polypropylene with calcium carbonate without FR additives showed two main steps of the decomposition temperature range. The first range of temperature is located from 323 °C to 493 °C, assigned with decomposition of polypropylene. The second step of decomposition temperature starts at 555 °C and ends at 723 °C, related to the calcium carbonate decomposition. These findings were also reported by Moaref et al. [39].

PP/CaCO_3_ with flame retardants showed three decomposition temperature ranges. For example, for the PP/CaCO_3_/MH5 composite, a slower degradation rate was observed from 309 °C to 409 °C. This was attributed to the dehydration of magnesium hydroxide. Yoshida et al. [40] and Genovese and Shanks [6] also reported the same behaviour within this range of temperature. Furthermore, the second decomposition was between 410 °C and 495 °C and it was associated with the effect of magnesium hydroxide into the PP/CaCO_3_ composite. Mg(OH)_2_ delayed the degradation of polypropylene. The third stage of decomposition was located approximately from 542 °C to 727 °C. This may be related to the interaction of magnesium hydroxide with calcium carbonate producing magnesium and calcium oxide residues that act as physical barriers to extend the degradation or reduce the decomposition of PP composite.

The same behaviour noted in the PP/CaCO_3_/MH5 composite was also observed in sample PP/CaCO_3_/MH10. However, 10 wt.% of magnesium hydroxide presented high thermal stability by delaying the maximum decomposition temperature of PP and had a slightly higher amount of char (33.9%), compared to the PP/CaCO_3_/MH5 which had 32.5% of char (Table 4). This demonstrated that high concentrations of magnesium hydroxide improved the degradation of the PP materials.

Samples of PP/CaCO_3_ with zinc borate also displayed three steps of decomposition. For example, in sample PP/CaCO_3_/ZB5, the first step of decomposition temperature range starts from 300 °C to 400 °C. Therefore, this may be ascribed to the dehydration of the zinc borate that forms zinc and boron oxides and water vapour. The second decomposition step is between 412 °C and 497 °C due to zinc borate in the PP/CaCO_3_, which produces zinc and boron oxides that help to delay the degradation rate of polypropylene. This improvement of the decomposition temperature of PP, with the addition of zinc borate, is higher than the one observed in the PP/CaCO_3_ composite. The third decomposition step starts at 579 °C and ends at 743 °C. The improved decomposition of PP with zinc borate and calcium carbonate when compared to the decomposition of PP with only calcium carbonate is due to the strong interaction of CaCO_3_ and ZB in the PP. This promotes the formation of pronounced char residues extending the thermal stability of PP materials.

It is important to note that at 700 °C, the glassy foam starts to be formed from zinc borate, therefore, in this study, the highest TGA temperature used was 900 °C. This was also justified by the high amount of char residue (31.3%) obtained for PP/CaCO_3_/ZB5 compared with char residue (28.1%) for the PP/CaCO_3_ composite (Table 4). As previously described in samples based on the PP/CaCO_3_ with magnesium hydroxide, the high concentration of zinc borate (10 wt.%) also affected the thermal stability of PP, demonstrated by improving the decomposition temperature of PP materials. With very high char (36.4%), it increased the degradation rate of PP.

The synergistic effect of zinc borate and magnesium hydroxide in the PP/CaCO_3_ composites was observed as the thermal stability of PP improved, as demonstrated by the highest range of decomposition temperature of PP/CaCO_3_ materials from 583 °C to 771 °C and had the highest char residue (47.2%) of all samples (Table 4). This may be justified by the good interaction of zinc borate and magnesium hydroxide, which resulted in the replacement of zinc with magnesium, suggesting that magnesium borate and zinc oxide are thermodynamically ideal chemicals for flame retardancy at elevated temperatures [6]. The reaction that describes the formation of zinc oxide by the reaction of dehydration of zinc borate and magnesium oxide formed by magnesium hydroxide [6] is presented below:

2ZnO·3B_2_O_3(s)_ + 2MgO_(s)_ → 2MgO·3B_2_O_3(s)_ + 2ZnO_(s)_

In general, it is expected that any boron oxide produced would be in a glassy, amorphous form. This glassy form is directly related to the high char residue obtained. Finally, the results clearly demonstrated that zinc borate also acts as an intumescent flame retardant. Together, with magnesium hydroxide, it formed a compact char residue capable of decreasing the heat and oxygen transmission and extending the degradation of PP, promoting the enhancement of thermal stability of PP-based composites.

### 3.5. Flame Retardancy

The limiting oxygen index (LOI) method is a crucial technique that provides quick and practical information, such as the flammability performance of the polymer materials, by evaluating the minimum concentration of oxygen needed in a mixture of oxygen and nitrogen gases to not burn the materials in ambient temperature. This flammability study is one of the requirements in the construction industry. The LOI data of neat PP and PP composites reinforced with flame retardants is reported in Table 4.

The filler and additives added in the polypropylene improved the LOI values to some level when compared to neat PP with an LOI value of 17.9%. For example, polypropylene with 50 wt.% of CaCO_3_ had 19.7% of LOI. This improvement in LOI value occurs because calcium carbonate acts as a gas-phase flame retardant, and during its decomposition, absorbs some of the combustion heat. This reduces the polymeric materials close to the flame by releasing the carbon dioxide (CO_2_) that reduces the gaseous reactants in the flame [41]. During the LOI measurement of neat PP, it was observed that the samples were easily ignited, and this sample burned vigorously and much faster than PP with flame retardants.

Zinc borate in the PP/CaCO_3_ composites also increased the LOI values. PP/CaCO_3_/ZB5 and PP/CaCO_3_/ZB10 composites presented 22.2% and 24.9% of LOI values, respectively. During thermal decomposition, ZB forms a glassy foam, and this covers the surface area of the polypropylene matrix, inhibiting the spread of flame and suppressing smoke. In addition, it was observed that a high amount of zinc borate increased the level of LOI.

A synergistic effect of zinc borate and magnesium hydroxide in the PP/CaCO_3_ composite was observed. Therefore, the LOI of PP/CaCO_3_/ZB10/MH10 was 29.4%. This may be explained by the good distribution and dispersion of ZB and MH in the polypropylene matrix that during thermal decomposition of flame retardants promoted the formation of an expanded dense carbon layer on the surface of PP material. This layer prevented the heat transfer between the external heat source and the matrix, isolating the evaporation of combustible gas on the outside of the sample, and interrupting further thermal degradation of the polymer. It is also important to note that zinc borate acts as an intumescent flame retardant, which corresponds to swelling and expansion of the polymer matrix beyond a critical temperature with the formation of a stable char structure. Furthermore, magnesium hydroxide releases water vapour and residue of magnesium oxide (MgO), which is capable of suppressing smoke and flame.

During the LOI test of PP/CaCO_3_ composites reinforced with zinc borate and magnesium hydroxide, some interesting observations were noted. For example, the samples were delayed for ignition, and, in a short period of time, the flame reduced and entirely stopped spreading, and the smoke was also suppressed.

In realistic fire conditions, products based on polymers are exposed to elevated temperatures. Therefore, to be considered self-extinguishing in a real fire condition, LOI values at ambient temperature must be high (the recommended minimum LOI value is 28%) [42]. In this study, the LOI values of PP/CaCO_3_/ZB10/MH10 composites (29.4%) satisfied the recommended LOI in a realistic situation. This LOI value is considered satisfactory for any application since materials used in the construction industry need to be self-extinguishing materials. John [43] also described that materials with LOI less than 21% are considered to be flammable, and LOI values greater than 21% are considered to be self-extinguishing. Therefore, PP/CaCO_3_/ZB5, PP/CaCO_3_/ZB10, PP/CaCO_3_/MH5, and PP/CaCO_3_/MH10 composites are considered to be self-extinguishing materials with LOI values greater than 21% (Table 4).

#### 3.5.1. Mechanism for Flame Retardancy Action and Synergistic Effect of ZB and MH in the PP

**Figure 6:**
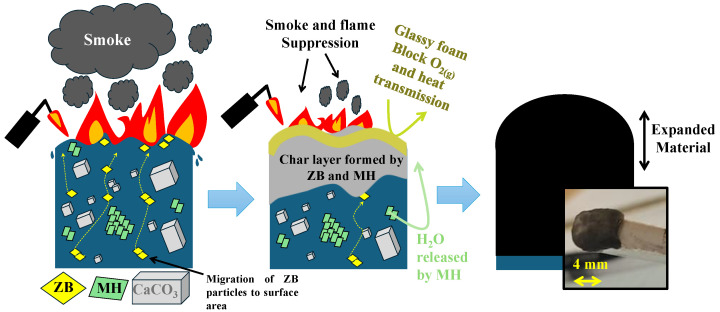
Proposed flame retardancy mechanisms for the system PP/CaCO_3_/ZB10/MH10 composites. The synergistic effect of zinc borate and magnesium hydroxide is demonstrated in this mechanism by blocking heat transmission and flame and smoke suppression.

The mechanism of flame retardancy and an exploration of the synergistic effect of zinc borate and magnesium hydroxide in the PP/CaCO_3_ composites is shown in Figure 6. This formulation presented the highest synergistic influence, demonstrated by the reduction of heat release and flame and smoke suppression with an LOI value of 29.4%. The mechanism proposed is based on four components: (i) At the beginning of the burning process of the PP/CaCO_3_/ZB10/MH10 composite, the polypropylene begins to melt and burn. (ii) Then, the particles of zinc borate migrate to the surface of the burning area of the polypropylene matrix, and the quantity of zinc borate particles migration increases to the surface area of PP. (iii) Next, the thermal decomposition of zinc borate and magnesium hydroxide takes place in the system of PP/CaCO_3_ composite. Several glassy foams are produced by zinc borate and these blow and completely cover the entire surface of the burning area of polymer, leading to rapid charring of PP chains from the surface to the inside, resulting in a compact barrier layer. The heating of magnesium hydroxide decomposes to a residue of magnesium oxide and water vapour through an endothermal process that enhances the ignition time [44]. Beyond the ignition time, the polypropylene creates a crosslinked network, and a carbonaceous char forms the boundary of PP degradation. The water vapour released from MH helps in flame and smoke suppression. (vi) At the end, the more pronounced and effective char layer obtained via the synergistic effect of zinc borate and magnesium hydroxide reduces the rate of degradation of the polypropylene, and consequently, there is a delay in the heat and oxygen transmission from the burning area to the non-degraded PP. The rate of degradation of the polypropylene behaviour was also explained by TGA results, where the high thermal stability of polypropylene with zinc borate and magnesium hydroxide was observed.

#### 3.5.2. Char Expansion Analysis of PP/CaCO_3_ Composites Reinforced with Flame Retardants

**Figure 7:**
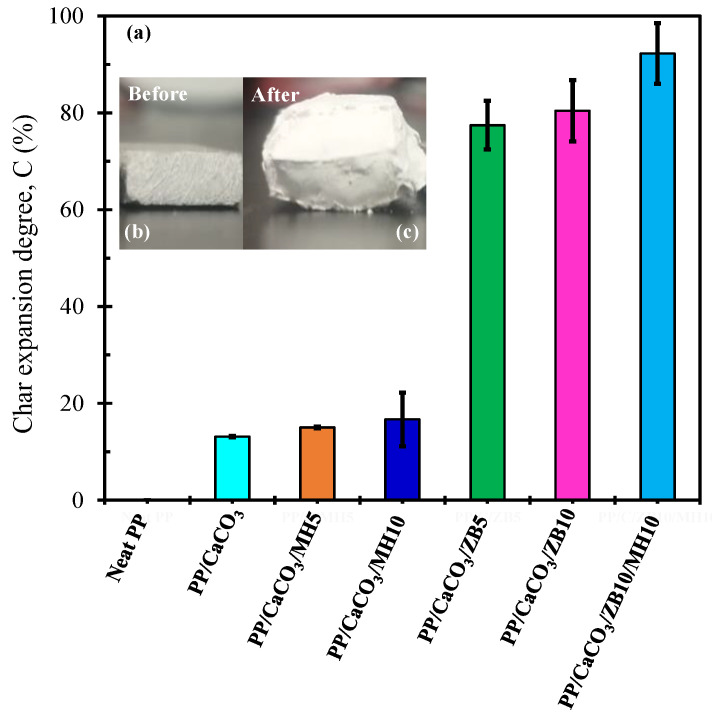
(**a**) Char expansion degree (C (%)) of PP/CaCO_3_ composites with flame retardants after the pyrolysis analysis at 900 °C for 20 min; exemplification of PP/CaCO_3_/ZB10/MH10 composite, (**b**) before, and (**c**) after char expansion of the sample.

The effect of inorganic flame retardants on the char expansion of PP/CaCO_3_ composites reinforced with additives was evaluated by pyrolysis test and the data is shown in Figure 7. The results demonstrated that the addition of zinc borate in the polypropylene/CaCO_3_ composites increased the char expansion. Additionally, it created a foamy structure on the char. This behaviour was also reported by Gillani et al. [32]. It was noted that a high content of ZB also increased the char expansion degree, and PP/CaCO_3_/ZB10/MH10 demonstrates the highest enhancement in char expansion. The char layer’s efficacy is greatly associated with its physical characteristics including foam structure and expansion [32,45].

A failure to establish an adhesive char network (or char expansion) was observed in samples without zinc borate, such as PP/CaCO_3_ and PP/CaCO_3_/MH composites. From the results, it is clear that boron oxide produced by zinc borate is associated with the intumescent behaviour of zinc borate, which plays a significant role in the char expansion of polymeric materials. The insert Figure 7b,c exemplifies the char expansion behaviour of PP/CaCO_3_/ZB10/MH10 after the pyrolysis test compared to the sample before the pyrolysis test. This is a clear example showing that adding zinc borate in the PP composite resulted in better char expansion, restricting gas growth in the PP matrix. The image in Figure 7c also demonstrates that the surface is compact enough and rigid enough to prevent heat transmission.

#### 3.5.3. Evaluation of the Mechanical Properties

**Figure 8:**
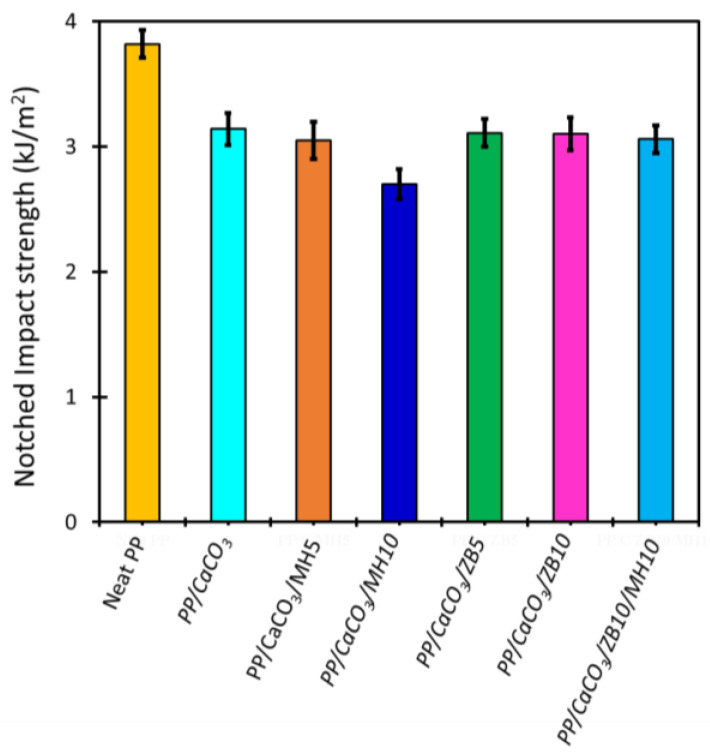
Impact strengths of neat PP and PP/CaCO_3_ composites with inorganic flame retardants.

The mechanical properties of neat PP and the composites were evaluated using tensile tests (Table 5) and Izod impact strength tests (Figure 8). The tensile test results are discussed in terms of three key properties, elastic modulus (E), ultimate tensile strength (UTS), and strain at break, which are crucial from an application standpoint. The addition of a high filler content (CaCO_3_) enhanced elastic modulus but adversely affected UTS and strain at break, as seen when compared with neat PP. Specifically, the elastic modulus increased with an increase in ceramic particle content, and composites containing 10% inorganic flame retardants exhibited a higher modulus than those with 5%. Notably, the PP/CaCO_3_/ZB10/MH10 composition achieved the highest modulus among the studied materials, with an increase in modulus exceeding 300% compared to neat PP.

This behaviour was anticipated, because adding stiff particles to a soft polymer matrix generally increases elastic modulus due to the high modulus of the ceramic particles (CaCO_3_). However, strain at break and UTS are highly dependent on the quality of the interfaces between polymer and filler (PP-CaCO_3_ or PP-flame retardants) [30]. Weak interfaces and high filler content, such as 50 wt.%, may act as stress concentrators within the polymer matrix, significantly reducing strain at break, and causing premature rupture. This also leads to a considerable decrease in UTS. This phenomenon is well documented in the literature and was also observed here, as strain at break values dropped significantly with the addition of fillers. UTS decreased by 30% in most composites compared to neat PP, indicating premature specimen fracture. The PP/CaCO_3_/ZB10/MH10 sample exhibited the highest modulus but had the lowest strain at break and a high standard error for UTS (nearly 25%), reinforcing that this highly filled composition is brittle. The tensile strength values obtained in this work are in agreement with the tensile strength values of 15.5–26.0 MPa for polypropylene/calcium carbonate (50 wt.%) materials found in the MatWeb database [46]. This demonstrates the efficiency of these materials to be used in the construction industry.

Similarly, the impact strength behaviour correlated negatively with the tensile test results. The addition of stiff ceramic particles in the polymer matrix led to a significant decrease in impact strength (Figure 8), particularly if the particle–matrix interface is weak. However, this decrease in impact was less pronounced than that observed for UTS and at strain at break, and most composites displayed impact strength values around 3 kJ/m^2^. Interestingly, the addition of 10 wt.% MH resulted in a further reduction in impact strength, potentially due to the morphological development of flatter-shaped particles in large aggregates, as seen in SEM images (Figure 3e). However, this reduction was not observed in the PP/CaCO_3_/ZB10/MH10 composite. Overall, the general reduction in impact strength does not significantly impact the material’s potential, especially for applications utilizing compositions similar to PP/CaCO_3_. Finally, it is important to note that the impact strength values around 3 kJ/m^2^ found in this work are in agreement with Izod impact strength (ISO) of 2.50–15.9 kJ/m^2^ for polypropylene/calcium carbonate (50 wt.%) materials found in the MatWeb database [46].

## 4. Conclusions

To develop polypropylene composites with high calcium carbonate and inorganic flame-retardant efficiency, which can be employed in the construction industry, high amount of calcium carbonate and concentrations of friendly inorganic additives varying from 5 wt.% to 10 wt.% were fabricated by melt mixing process using a batch mixer. The influence of flame retardants and their concentrations on the processability, thermal, mechanical, and flame-retardant properties of PP was investigated. The obtained results revealed that the optimum composite of PP/CaCO_3_/ZB10/MH10 offered a better improvement in composite properties. For this composite, the maximum thermal degradation temperature increased from 294 °C to 771 °C compared to neat PP and other PP composites. This showed that a significant interaction between MH and ZB delayed the degradation of PP and calcium carbonate. This composite showed a high LOI value of 29.4%, and a satisfactory char residue of 47.2%. Also, the flame retardants and calcium carbonate did not prejudice the mechanical properties, such as tensile strength and impact strength of the PP materials, demonstrating that these values are in agreement with the values reported in the literature. SEM analysis showed that the presence of additives and calcium carbonate resulted in reasonable compatibility between themselves and between PP composites and flame retardant additives. Finally, it can be concluded that PP/CaCO_3_/ZB10/MH10 is the most promising flame-retardant system in terms of enhanced thermal stability, with favourable mechanical properties, good processability, and better flame retardancy, providing smoke and afterglow suppressant, self-extinguishing, and anti-dripping properties. As a result, these outstanding properties make the PP/CaCO_3_/ZB10/MH10 an environmentally friendly, halogen-free flame-retardant polymer composite with great potential for industrial applications including the construction industry.

## Figures and Tables

**Table 1:** Neat PP and different formulations of PP based on CaCO_3_ composites containing inorganic flame retardants.

Samples	PP (wt.%)	CaCO_3_ (wt.%)	ZB (wt.%)	MH (wt.%)
Neat PP	100	0	0	0
PP/CaCO_3_	50	50	0	0
PP/CaCO_3_/MH5	45	50	5	0
PP/CaCO_3_/MH10	40	50	10	0
PP/CaCO_3_/ZB5	45	50	0	5
PP/CaCO_3_/ZB10	40	50	0	10
PP/CaCO_3_/ZB10/MH10	30	50	10	10

**Table 2:** Power law fit parameters of the viscosity of the composites.

Samples	Intercept (Log m)	Slope (n − 1)	Adjusted-R^2^	Consistence Index (m) 10^1^ (Pa.s^n^)	Pseudoplasticity Index (n)
Neat PP	3.24 ± 0.02	−0.59 ± 0.01	0.998	174 ± 72	0.41 ± 0.01
PP/CaCO_3_	2.54 ± 0.04	−0.4 ± 0.02	0.984	35 ± 3	0.60 ± 0.02
PP/CaCO_3_/MH5	2.53 ± 0.08	−0.39 ± 0.04	0.931	33 ± 6	0.61 ± 0.04
PP/CaCO_3_/MH10	2.77 ± 0.04	−0.43 ± 0.02	0.982	58 ± 6	0.57 ± 0.02
PP/CaCO_3_/ZB5	2.71 ± 0.04	−0.43 ± 0.02	0.983	51 ± 5	0.57 ± 0.02
PP/CaCO_3_/ZB10	2.73 ± 0.05	−0.42 ± 0.02	0.977	54 ± 6	0.58 ± 0.02
PP/CaCO_3_/ZB10/MH10	2.97 ± 0.05	−0.48 ± 0.02	0.984	92 ± 10	0.52 ± 0.02

**Table 3:** DSC parameters of neat PP and PP-based CaCO_3_ composites with flame retardants.

Samples	T_m_ (°C)	ΔH_m_ (J/g)	X_c_ (%)	Tc (°C)	ΔH_c_ (J/g)	T_m_ (°C)	ΔH_m_ (J/g)	X_c_ (%)
Neat PP	166	87	42	109	99	162	91	44
PP/CaCO_3_	165	86	42	119	52	160	91	44
PP/CaCO_3_/MH5	162	83	40	117	46	159	89	43
PP/CaCO_3_/MH10	165	80	39	119	38	161	85	41
PP/CaCO_3_/ZB5	165	81	39	120	43	165	82	40
PP/CaCO_3_/ZB10	162	84	40	119	39	161	87	42
PP/CaCO_3_/ZB10/MH10	164	68	33	118	26	159	73	35

T_m_—melting temperature; ∆H_m_—melting enthalpy; T_c_—crystallization temperature; ∆H_c_—crystallization enthalpy; and X_c_—crystallinity degree.

**Table 4:** TGA and LOI results of neat PP and PP-based CaCO_3_ composites with inorganic flame retardants.

Samples	First Stage of Temperature (°C)	Second Stage of Temperature (°C)	Third Stage of Temperature (°C)	Char Residue (%)	LOI (%)
Neat PP	N/A	340–480	N/A	0.0	17.9
PP/CaCO_3_	N/A	323–493	555–723	28.1	19.7
PP/CaCO_3_/MH5	309–409	410–495	542–727	32.5	21.2
PP/CaCO_3_/MH10	334–413	414–500	579–752	33.9	23.1
PP/CaCO_3_/ZB5	300–400	412–497	579–743	31.3	22.2
PP/CaCO_3_/ZB10	304–400	404–500	542–757	36.4	24.9
PP/CaCO_3_/ZB10/MH10	294–409	410–472	583–771	47.2	29.4

**Table 5:** Summarized tensile mechanical properties of neat PP and its composites, in terms of mean ± standard deviation.

Samples	Elastic Modulus (MPa)	Ultimate Tensile Strength (MPa)	Strain at Break (%)
Neat PP	1158 ± 18	31.4 ± 0.9	6.0 ± 1.0
PP/CaCO_3_	2427 ± 156	23.4 ± 1.1	1.5 ± 0.2
PP/CaCO_3_/MH5	2610 ± 260	21.2 ± 0.6	1.2 ± 0.2
PP/CaCO_3_/MH10	3095 ± 102	22.3 ± 0.6	0.9 ± 0.1
PP/CaCO_3_/ZB5	2446 ± 136	19.7 ± 0.3	1.0 ± 0.1
PP/CaCO_3_/ZB10	2989 ± 241	20.0 ± 0.9	1.5 ± 0.2
PP/CaCO_3_/ZB10/MH10	4804 ± 438	20.1 ± 6.0	0.6 ± 0.2

## Data Availability

The data presented in this study are available on request from the corresponding author.
